# Molecular Doping Induced Charge Transfer Complex Formation and Interfacial Dopant Interdiffusion on Graphite

**DOI:** 10.1021/acs.jpcc.5c05680

**Published:** 2025-12-04

**Authors:** Christos Gatsios, Andreas Opitz, Patrick Amsalem, Thorsten Schultz, Remy Jouclas, Yves Geerts, Norbert Koch

**Affiliations:** † Institut für Physik & Center for the Science of Materials Berlin (CSMB), 9373 Humboldt-Universität zu Berlin, 12489 Berlin, Germany; ‡ Helmholtz-Zentrum Berlin für Materialien und Energie GmbH, 12489 Berlin, Germany; § Laboratoire de Chimie des Polymères, Faculté des Sciences, Université Libre de Bruxelles (ULB), Boulevard du Triomphe, CP 206/01, Bruxelles 1050, Belgium; ∥ International Solvay Institutes for Physics and Chemistry, Université Libre de Bruxelles (ULB), Boulevard du Triomphe, CP 231, Bruxelles 1050, Belgium

## Abstract

Doping is a powerful method to optimize the electrical characteristics of organic semiconductors, but a comprehensive picture capturing the phenomena at play is still under development. In this work, combining UV–vis absorbance spectroscopy with ultraviolet and X-ray photoelectron spectroscopy, we investigate the p-type doping of (sub)­monolayer films of two structurally isomeric organic semiconductors on graphite, naphtho­[2,3-*b*]­thieno-[2‴,3‴:4″,5″]­thieno­[2″,3″:4′,5′]­thieno­[3′,2′-b]­naphtho­[2,3-*b*]­thiophene (DN4T) and naphtho­[1,2-*b*]­thieno­[2‴,3‴:4″,5″]­thieno­[2″,3″:4′,5′]­thieno­[3′,2′-b]­naphtho­[1,2b]­thiophene (isoDN4T), with the strong molecular acceptor 2,2′-(perfluoronaphthalene-2,6-diylidene)­dimalononitrile (F6TCNNQ). For DN4T, a hybrid highest occupied molecular level emerges from the hybridization of the DN4T and F6TCNNQ frontier occupied levels, resulting from the formation of DN4T:F6TCNNQ charge-transfer complexes. With increasing F6TCNNQ coverage, the electronic levels of both neutral DN4T and the DN4T:F6TCNNQ complexes shift toward the Fermi level because of an interface dipole that is due to electron transfer from graphite to F6TCNNQ. In comparison, isoDN4T exhibits stronger interaction with F6TCNNQ and increased interfacial disorder, as evidenced by significant spectral broadening. These findings emphasize the profound impact of subtle structural variations on host–dopant interactions and the importance of exploring multicomponent interfaces for advanced organic electronic and optoelectronic applications.

## Graphical abstract

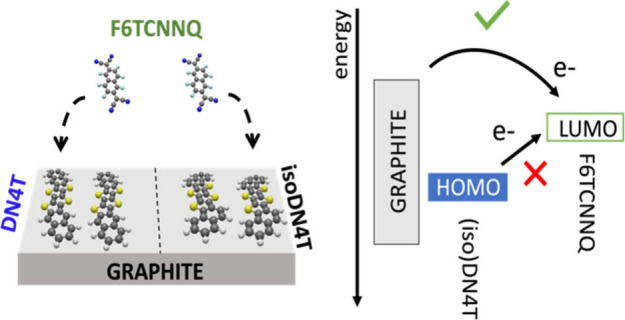

## 1 Introduction

Molecular doping of organic semiconductors presents an effective strategy to enhance the performance and stability of organic-based devices. Key benefits include improved electrical conductivity, improved thermal and environmental stability, and the ability to intentionally modify the electronic properties of organic semiconductors by forming new hybrid states within the energy gap.^1–5^ The relatively large size of molecular dopants facilitates controllable processing, and reduces the likelihood of interdiffusion and Coulombic interactions with charge carriers.^6–10^ However, a comprehensive picture of molecular dopant interactions with the host organic semiconductors, the thermodynamic limitations of doping, as well as the effects on charge transport still needs further development.^4^

Molecular doping is achieved by introducing electron-accepting (p-type) or electron-donating (n-type) molecules into the organic semiconductor matrix. Solution-based or physical vapor deposition techniques are typically used for doping.^11–15^ At the molecular scale, doping is understood as a charge transfer process between the dopant and host molecule. Depending on the system, this transfer may involve either an integer charge, leading to ion-pair formation, or partial charge transfer with a significant degree of orbital hybridization between the dopant and host.^16,17^ As a rule of thumb, ion-pair formation is favorable when the frontier energy levels of the dopant lie sufficiently close to those of the host molecule. For example, p-doping is energetically favored when the lowest unoccupied molecular orbital (LUMO) level of the dopant lies below the highest occupied molecular orbital (HOMO) level of the semiconductor molecule, i.e., the electron affinity (EA) of the dopant is higher than the ionization energy (IE) of the host semiconductor. However, solid-state interactions often modify the energy levels of both the dopant and host molecule, complicating this picture. For instance, previous theoretical studies have shown that intermolecular arrangements and electrostatic interactions via molecular quadrupoles can significantly shift orbital energies.^18–20^ Furthermore, the structural and energetic disorder in molecular systems adds significant complexity in predicting the outcomes of molecular doping. Experimental and theoretical studies suggest that the resulting electronic properties in these multicomponent materials require a refined consideration of the dopant structure, as well as a deeper understanding of the host–dopant interactions and thermodynamics of doping.^4,21^

Dopant structure is critical in controlling and optimizing charge transfer. For example, tetracyanoquinodimethane (TCNQ) derivatives like 2,3,5,6-tetrafluoro-7,7,8,8-tetracyanoquinodimethane (F4TCNQ) and 2,2′-(perfluoronaphthalene-2,6-diylidene)­dimalononitrile (F6TCNNQ) have been engineered as potent electron acceptors. Adding fluorine atoms to TCNQ increases its EA, making it a stronger p-type dopant.^22,23^ Additionally, extending the aromatic core in F6TCNNQ improves thermal and morphological stability, which are essential for long-term device performance.^24,25^ However, F6TCNNQ can alter the electronic properties of host semiconductor molecules in unintended ways by forming charge transfer complexes (CTCs).^26,27^ These complexes correspond to the formation of host-dopant dimers whose frontier molecular orbitals hybridize as illustrated in Figure 1, resulting in a new set of electronic states very sensitive to the fine details of the intermolecular interaction. This underscores the need for further investigations into how dopants interact with various organic semiconductors.

**Figure 1:**
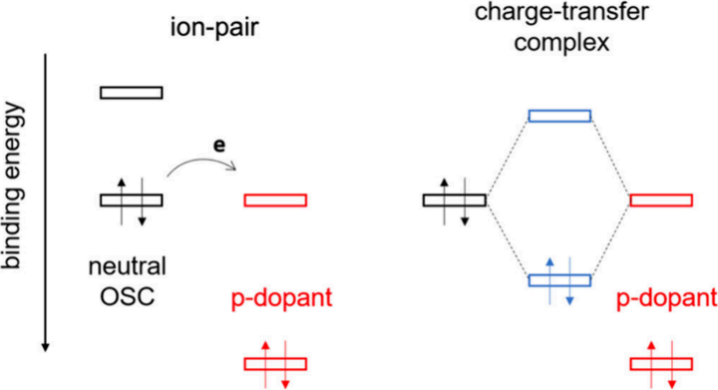
Two cases of host–dopant interactions. Left: integer charge transfer resulting in the formation of ion-pairs. Right: charge transfer complex formation resulting in a new pair of frontier hybrid orbitals of the complex.

Increasing doping efficiency requires not only optimizing the dopant structure and host–dopant interactions but also carefully engineering the energy level alignment in complex systems. This can be particularly difficult to apprehend as molecularly doped organic systems often involve not just host semiconducting molecules and molecular dopants, but also charge-transfer complexes, a material substrate and additional defects. In such cases, doping is governed by the final position of the chemical potential of the electrons (Fermi level) of the entire system, relative to the density of states of the organic semiconductor.^16,28^ For these reasons, exploring a diversity of such systems and analyzing how the energy level alignment is achieved in multicomponent interfaces should help understanding and improving device performance.

Previous work explored two isomeric molecular semiconductors: naphtho­[2,3-*b*]­thieno-[2‴,3‴:4″,5″]­thieno­[2″,3″:4′,5′]­thieno­[3′,2′-b]­naphtho­[2,3-*b*]­thiophene (DN4T) and naphtho­[1,2-*b*]­thieno­[2‴,3‴:4″,5″]­thieno­[2″,3″:4′,5′]­thieno­[3′,2′-b]­naphtho­[1,2b]­thiophene (isoDN4T).^29,30^ Despite their similar structures and packings, it was shown that hole-vibration coupling is stronger in isoDN4T and the transfer integrals between isoDN4T molecules smaller compared to DN4T, which makes DN4T a more efficient hole transport semiconductor. Considering all the above, our current work investigates the molecular doping of DN4T and isoDN4T thin films with F6TCNNQ, a strong molecular acceptor. Although several studies already exist discussing the molecular doping of classic organic semiconductors such as pentacene, tetracene, and P3HT with F6TCNNQ and its structurally similar F4TCNQ,^17,31,32^ direct photoelectron spectroscopy investigations of emerging thienoacenes like DN4T and isoDN4T remain scarce. Moreover, there are few works on the structurally related DNTT (dinaphtho­[2,3-b:2′,3′-f]­thieno­[3,2-*b*]­thiophene) doped with F4TCNQ and F6TCNNQ that demonstrate device performance improvements,^33–35^ underscoring the need for a deeper understanding of host–dopant interactions in these systems and their impact on electronic energy levels.

DN4T and isoDN4T layers were deposited via thermal sublimation in ultrahigh vacuum on highly oriented pyrolytic graphite (HOPG), the surface of which may be considered equivalent to that of graphene, a technologically relevant material as flexible and transparent electrode.^36–38^ Importantly, the inertness of the HOPG surface allowed us to isolate and distinguish host–dopant interactions. The electronic structure of the doped thin films was investigated *in situ* by ultraviolet photoelectron spectroscopy (UPS) as well as X-ray photoelectron spectroscopy (XPS), and UV–vis absorbance spectroscopy was employed *ex-situ* to detect the optical signatures of ion-pair or charge-transfer complex formation. Although the experimental conditions necessarily differ, UV–vis absorbance requires thicker films on transparent substrates, whereas UPS/XPS probe only the topmost molecular layers and are ideally performed on conductive substrates, their complementary use provides a consistent picture of CTC or ion-pair formation.^16,26,32^ This is because the underlying intermolecular electronic interactions between host and dopant can manifest both in bulk and at interfacial mixtures.

When F6TCNNQ was deposited onto the DN4T layers, we resolve unequivocally the spectral signature of DN4T:F6TCNNQ charge-transfer complex formation. The binding energy position of the new hybrid HOMO level of the complex further indicates that the LUMO level of the complexes is pinned relative to the Fermi level. Interestingly, the electronic levels of both neutral DN4T and DN4T:F6TCNNQ complexes shift toward the Fermi level with increasing F6TCNNQ coverage. This shift is attributed to a change in the surface electrostatic potential, dominated by F6TCNNQ interdiffusion inducing direct electron transfer from HOPG to F6TCNNQ. As a result, DN4T develops a stronger p-type character. In the case of isoDN4T, the significant broadening of the photoelectron spectra, taken together with the stronger CTC absorbance features, points to a comparatively stronger CTC formation accompanied by abrupt surface morphological rearrangements. These, in turn, give rise to increased and spatially homogeneous electrostatic disorder.

Our UPS findings complemented by UV–vis absorbance data highlight a special case of energy-level alignment at the DN4T–F6TCNNQ–HOPG three-component interface, where charge-transfer complex formation, dopant interdiffusion, and interfacial charge transfer coexist and can be rationalized by straightforward electrostatic considerations, a scenario that has been reported only in few prior works.^39,40^ Comparison with the isomeric isoDN4T–F6TCNNQ–HOPG system further underscores how subtle structural variations can strongly influence intermolecular interactions. We therefore believe that our results provide a valuable contribution to the understanding of complex host–dopant interactions on graphite surfaces and their impact on energy-level alignment.

## 2 Materials and Methods

### 2.1 Sample Preparation

DN4T and isoDN4T, shown in Figure 2, were synthesized as per the methodology outlined in previous work.^29^ The IE of both DN4T and isoDN4T measured here and in the previous study was 6.2 eV.^30^ The molecular acceptor, F6TCNNQ, also shown in Figure 2, was purchased from Novaled GmbH. F6TCNNQ has an EA of 5.6 eV taken from a previous inverse photoelectron spectroscopy study.^25^ All molecules were subjected to thermal sublimation under ultrahigh vacuum (UHV) conditions (10^–8^ mbar) using resistively heated quartz crystal crucibles. We maintained a nominal deposition rate of 0.1 Å/min for all molecules, a rate determined using a quartz crystal microbalance and based on an assumed molecular density of 1.3 g/cm^3^. The highly oriented pyrolytic graphite (HOPG) substrates were first cleaved in ambient conditions and then annealed at 350 °C in UHV for 1 h prior to the molecular deposition, in order to desorb unwanted adsorbates.

**Figure 2:**
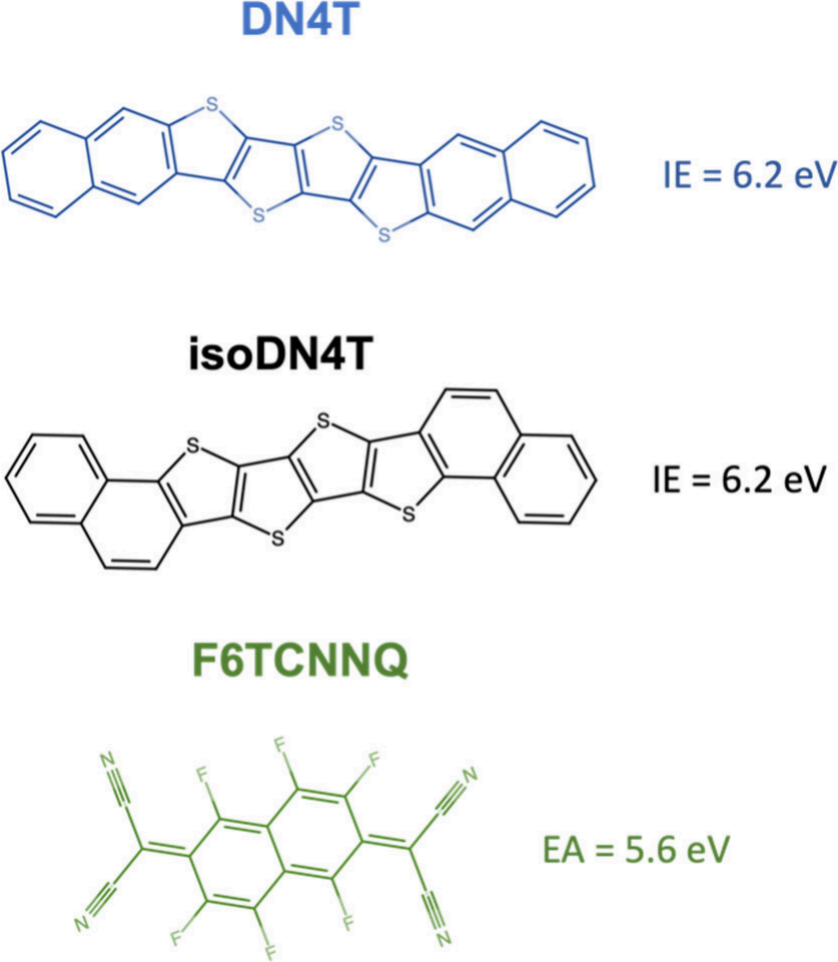
Molecular structure of DN4T, isoDN4T, and the molecular acceptor F6TCNNQ.

### 2.2 Photoelectron Spectroscopy

Ultraviolet and X-ray photoelectron spectroscopy measurements (UPS and XPS) were conducted using a hemispherical analyzer system (SPECS PHOIBOS 100) in an analysis chamber with 2 × 10^–10^ mbar base pressure. For UPS, we utilized a He discharge lamp (HIS13, ScientaOmicron) equipped with a VUV monochromator, operating at an excitation energy of 21.22 eV. We recorded the valence spectra with a pass energy of 5 eV, achieving an energy resolution of 76 meV. The secondary electron cutoff (SECO) region was examined by applying a −10 V bias and using a pass energy of 2 eV, resulting in a 62 meV resolution. We performed additional UPS and XPS measurements at the ENERGIZE endstation at BESSY II, maintaining a base pressure of 1.8 × 10^–10^ mbar in the analysis chamber. For these experiments, we used a DA30L analyzer from ScientaOmicron. The pass energies were set to 50 eV to obtain the core levels in XPS, 5 eV for the valence levels, and 2 eV for the SECO. A DAR400 X-ray source from ScientaOmicron with a magnesium anode (*h*ν = 1253.6 eV) was used for XPS excitation, while a HIS13 He discharge lamp from ScientaOmicron was employed for the UPS measurements. These settings yielded energy resolutions of 980 meV for XPS and 70 meV for UPS. We applied a bias of −10 V to the sample during SECO measurements. Calibration of the binding energy scale involved measuring the Fermi edge of a clean Ag(111) surface, setting the center of the Fermi edge to 0 eV. Spectra analysis was conducted using Igor Pro 9 software (Wavemetrics).

### 2.3 UV–vis Absorbance Spectroscopy

Optical absorbance measurements were conducted in ambient conditions with a Lambda 950 UV/vis/NIR spectrophotometer from PerkinElmer Inc. For these measurements, thin films of DN4T, isoDN4T, F6TCNNQ and coevaporated DN4T:F6TCNNQ and isoDN4T:F6TCNNQ were deposited on quartz crystal substrates via thermal evaporation in UHV. During coevaporation both molecular sources were heated simultaneously until each of them achieved a nominal deposition rate of 0.1 Å/s. The intended molecular ratio of DN4T/isoDN4T to F6TCNNQ was 1:1, and the final thickness of the coevaporated films was 15 nm.

## 3 Results and Discussion

### 3.1 Optical Absorbance Spectra of DN4T:F6TCNNQ and isoDN4T:F6TCNNQ Mixtures: Evidence of Intermolecular Hybridization

Figure 3a and 3b show the absorbance spectra of pristine thin films of DN4T, isoDN4T, F6TCNNQ, and their mixtures thermally codeposited on quartz glass. The spectra for the individual compounds (DN4T, isoDN4T, and F6TCNNQ) are consistent with previously reported results.^24,29,41^ For the DN4T:F6TCNNQ mixture in Figure 3a, the absorbance spectrum resembles a superposition of the individual DN4T and F6TCNNQ spectra. However, an additional absorbance peak emerges at low photon energy, with absorbance maximum at 0.87 eV and onset at 0.71 eV. In the isoDN4T:F6TCNNQ mixture, a similar effect is observed, but significantly more pronounced. Notably, the new absorbance peak, with maximum at 0.98 eV and onset at 0.71 eV, becomes much more prominent compared to DN4T:F6TCNNQ, at the expense of the F6TCNNQ absorbance features.

**Figure 3:**
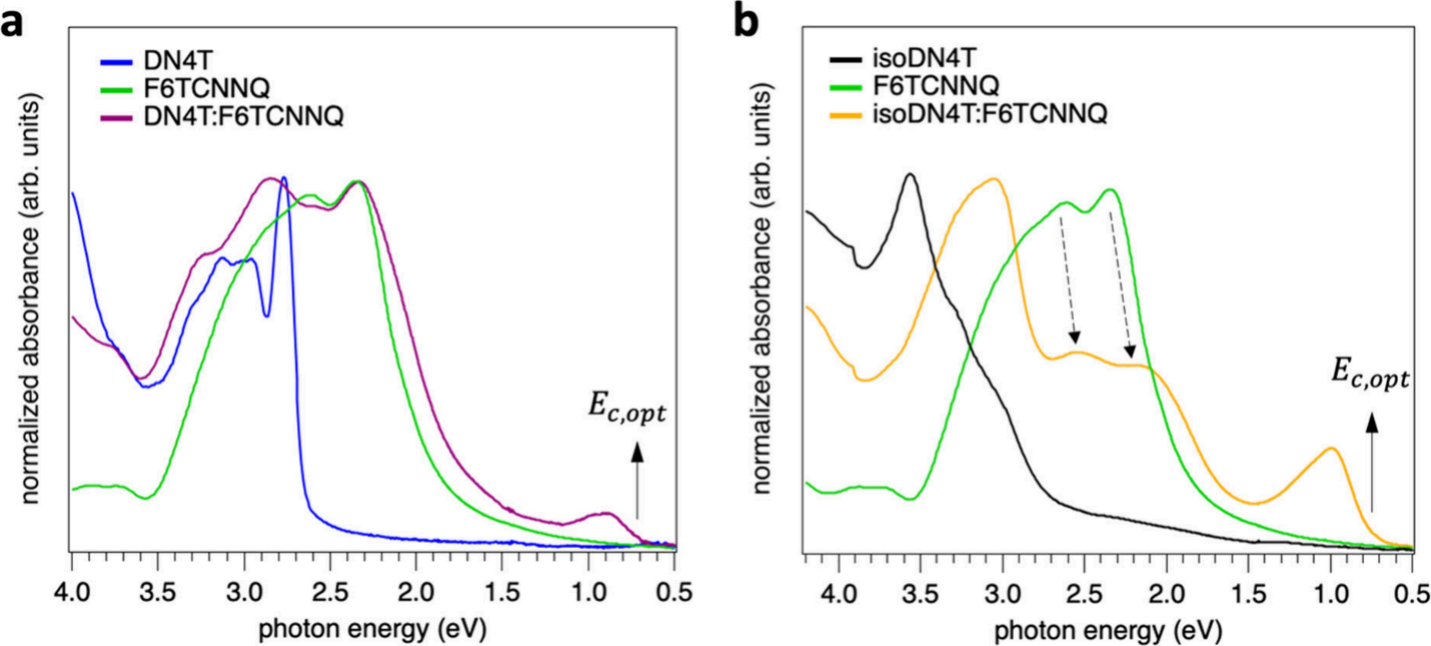
Optical absorbance spectra for pristine DN4T, isoDN4T, and F6TCNNQ thin films, as well as coevaporated mixtures of DN4T:F6TCNNQ (panel a) and isoDN4T:F6TCNNQ (panel b) deposited on quartz glass at a 1:1 molecular ratio. Solid arrows mark the onset of new optical transitions, attributed to the optical gaps of charge-transfer complexes. Dashed arrows highlight the pronounced decrease in F6TCNNQ absorbance features, likely reflecting a higher concentration of F6TCNNQ:isoDN4T complexes compared to unreacted F6TCNNQ molecules.

The observed low-energy optical transitions do not resemble the characteristic absorbance signatures of F6TCNNQ anions, which exhibits a sharp transition with a series of vibronic progressions between 1–1.3 eV, as reported in previous studies.^42,43^ This suggests that integer charge transfer leading to ion-pair formation is not the dominant process in the present cases and, instead, the formation of CTCs appears more plausible. In fact, several works have reported the formation of CTCs between F6TCNNQ and other planar conjugated molecules.^26,41,44^ Such complexes are expected to form due to wave function overlap between the LUMO of F6TCNNQ and the HOMO of DN4T (or isoDN4T), resulting in new bonding and antibonding hybrid electronic states with a smaller energy separation (HOMO–LUMO gap of the complex) compared to the HOMO–LUMO gap of the pristine DN4T (or isoDN4T) thin films (cf. Figure 1). For this reason, the low-energy absorbance peaks can be attributed to optical transitions corresponding to the HOMO–LUMO gap of the complexes.

Importantly, in the isoDN4T:F6TCNNQ mixture, the strong suppression of the F6TCNNQ absorbance features, combined with the enhanced intensity of the new absorbance peak, indicates a more favorable interaction between F6TCNNQ and isoDN4T compared to DN4T. This stronger interaction is likely facilitated by the enhanced wave function overlap related to the molecular structure of isoDN4T. As a result, the probability of charge-transfer complex formation, and therefore the concentration of the complexes, is significantly higher in the isoDN4T:F6TCNNQ mixture than in the DN4T:F6TCNNQ mixture.

### 3.2 UPS Analysis and Energy Level Shifts

Figure 4a and 4b demonstrate the evolution of the secondary electron cutoff (SECO) and valence electronic levels of DN4T layers on HOPG, upon subsequent deposition of F6TCNNQ. At a nominal thickness of 2 Å DN4T, corresponding to submonolayer-to-monolayer coverage as demonstrated from scanning tunnelling microscopy images in our previous work,^30^ DN4T exhibits a sharp HOMO peak at 1.66 eV binding energy, labeled H_DN4T_. After depositing 2 Å and 5 Å of F6TCNNQ, a new spectral feature (H_C_) emerges at the lower binding energy side of the H_DN4T_, peaking at 1.3 eV. Concomitantly, the sample work function remains almost constant at ca. 4.6 eV. Therefore, H_C_ appears to be a feature of DN4T in contact with F6TCNNQ: (i) it is not due to the F6TCNNQ HOMO, which lies at much higher binding energy; (ii) it does not stem from F6TCNNQ anions since the sample work function remains constant (signaling the absence of interface dipoles resulting from charge transfer) and their spectral signature, consisting of two features near the Fermi level, are not observed. Therefore, and consistent with the UV–vis measurements, this suggests that H_C_ represents the HOMO of F6TCNNQ:DN4T CTCs formed upon the early F6TCNNQ deposition steps and coexisting with “pristine”, nonreacted DN4T molecules. Moreover, the introduction of F6TCNNQ primarily affects the valence region of the HOMO with the appearance of H_C_, while the deeper electronic levels are much less perturbed. This observation is in line with wave function overlap between the HOMO of DN4T and the LUMO of F6TCNNQ, which should perturb only the outermost electronic levels. The large “uphill” energy difference between the DN4T HOMO and F6TCNNQ LUMO for electron transfer (cf. Figure 4c) makes ion-pair formation highly unlikely, indeed strongly suggesting the formation of a ground-state DN4T:F6TCNNQ CTC.

**Figure 4:**
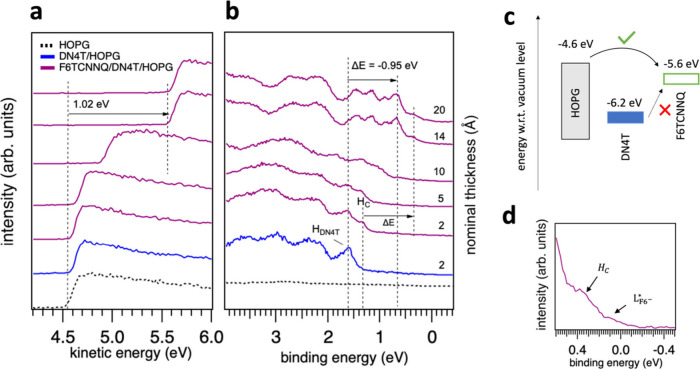
UPS spectra and energy level diagram for DN4T upon successive F6TCNNQ deposition on HOPG. (a) SECO and (b) valence region spectra, recorded at an off-normal emission angle of 45°. The dashed black curve corresponds to the HOPG substrate, while the blue curve represents 2 Å of DN4T deposited on HOPG corresponding to (sub)­monolayers of DN4T. The purple curves show the evolution of the DN4T spectra after successive F6TCNNQ depositions, highlighting the emergence of the new spectral feature (H_C_) and the uniform shift of the valence levels toward lower binding energies. (c) Energy level diagram of HOPG, DN4T, and F6TCNNQ relative to the vacuum level. (d) Magnified view of the 14 Å F6TCNNQ spectrum in the valence region, indicating the HOMO level of F6TCNNQ:DN4T CTC (H_C_), and the relaxed LUMO (L_F6^–^_^*^) associated with F6TCNNQ anions.

In contrast, further F6TCNNQ depositions uniformly shift the valence levels to lower binding energies, with the H_C_ peak remaining at fixed position relative to the H_DN4T_ peak. This is now accompanied by a strong work function increase by about 1 eV to ca. 5.55 eV when going from 5 Å to 14 Å nominal F6TCNNQ coverage and beyond. This behavior suggests that the DN4T molecules and the CTCs remain charge-neutral and are only shifted due to a change in the electrostatic landscape related to other charge transfer processes at the interface. In Figure 4c, we evaluate the likelihood of electron transfer processes by considering the relative positions of the frontier electronic levels of DN4T, F6TCNNQ, and HOPG. As schematically illustrated, IE of DN4T on HOPG (6.2 eV) is 0.6 eV below the EA of F6TCNNQ (5.6 eV). This significant energy barrier of 0.6 eV between the HOMO of DN4T and LUMO of F6TCNNQ makes electron transfer highly unlikely. However, given the lower work function of HOPG (4.6 eV), electron transfer from the valence states of HOPG to F6TCNNQ is energetically favorable. This can be verified at nominal F6TCNNQ thicknesses exceeding 10 Å, where the signature of F6TCNNQ anions in the photoelectron spectra appear as two additional features just below the Fermi level as displayed in Figure S1. These have been previously assigned to the relaxed HOMO and LUMO levels of F6TCNNQ in its anionic form, with peaks at 0.62 and 0.03 eV, respectively.^43,45,46^ Figure 4d shows evidence of F6TCNNQ anions particularly the relaxed LUMO level of F6TCNNQ (now partially filled), which appears as a state right above the Fermi level at approximately 0.03 eV. In addition, the SECO shift of 0.92 eV and the final work function of 5.55 eV observed for F6TCNNQ/HOPG (Figure S1, SI) match the one of F6TCNNQ/DN4T/HOPG, supporting electron transfer from HOPG to F6TCNNQ as the dominant underlying process.

### 3.3 Morphology of the F6TCNNQ/DN4T/HOPG Interface

The deeper electronic levels of DN4T shift consistently toward lower binding energies upon F6TCNNQ deposition. As shown in Figure 5a, shifting the pristine DN4T spectrum by −0.95 eV yields good agreement with the spectrum obtained after 14 Å of F6TCNNQ deposition, even for electronic states above 1.8 eV. This shift is also evident in the core levels. For example, the S 2p core level (Figure 5b) exhibits a slightly smaller shift of −0.75 eV. This observation could imply an adsorption morphology, where the DN4T layer is situated between the HOPG substrate and F6TCNNQ, analogous to a dielectric positioned in a parallel-plate capacitor, as shown in Figure 6a. After charge transfer from HOPG to F6TCNNQ, HOPG behaves as the positive plate, while the F6TCNNQ anion layer functions as the negative plate. As reported in previous studies,^39,46–48^ a linear potential gradient then forms between HOPG and the F6TCNNQ anion layer, creating a constant electric field experienced by the DN4T molecules. The total change in electrostatic potential at the F6TCNNQ surface corresponds to the observed work function change of 1.1 eV. In this scenario, the electronic levels of the DN4T dielectric layer reside in a shifted electrostatic potential, but the energy level shift should be significantly lower than the work function change (ca. half of it in the simple model of Figure 6).

**Figure 5:**
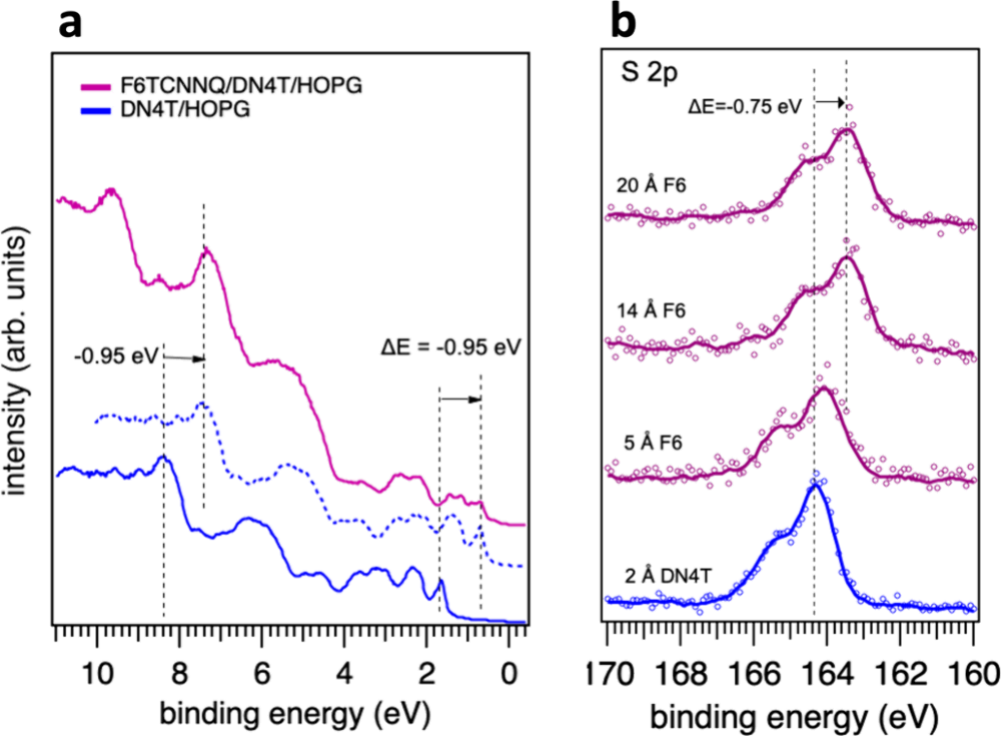
Survey valence spectra (a) and spectra of the S 2p core level of DN4T (b), indicating an energy shift associated with the charge rearrangement at the interface due to the introduction of F6TCNNQ. The blue solid curve shows the as measured spectrum of DN4T. The blue dashed curve corresponds to the valence spectrum of DN4T shifted by −0.95 eV, to allow comparison with the final purple spectrum of the F6TCNNQ/DN4T/HOPG system.

**Figure 6:**
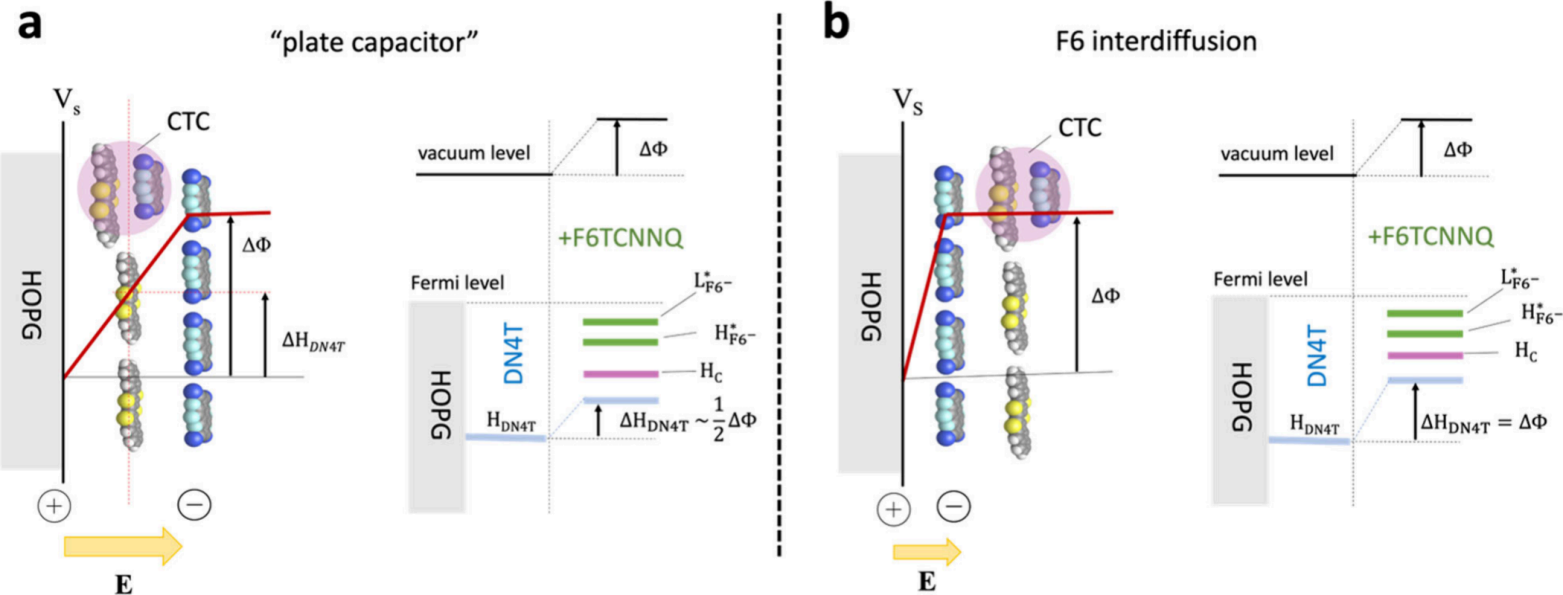
Qualitative illustration of two different adsorption configurations for the F6TCNNQ/DN4T/HOPG interface and the resulting electrostatic potential changes induced by electron transfer from HOPG to F6TCNNQ. In both configurations, electron transfer from HOPG to F6TCNNQ generates a negative charge localized on the F6TCNNQ layer and a compensating positive charge in HOPG, establishing a linear potential gradient. In configuration (a), which resembles a parallel-plate capacitor, F6TCNNQ is deposited on top of the DN4T layer. The resulting surface potential (*V*_S_) induces a vacuum level shift (ΔΦ) and an internal electrostatic potential change within DN4T, approximately half of the vacuum level shift (Δ*H*_DN4T_ ∼ ^1^/_2_ΔΦ). In configuration (b), F6TCNNQ interdiffuses beneath the DN4T layer, leading to a uniform shift of both the electronic levels and the vacuum level across the DN4T and the charge transfer complex (CTC), such that Δ*H*_DN4T_ = ΔΦ.

However, as explained in the following, we favor another adsorption morphology, also illustrated in Figure 6b. This involves interdiffusion of F6TCNNQ between the HOPG substrate and the DN4T layer, a situation that has been reported for the structurally similar F4TCNQ molecule and the large HATCN molecule.^39^ In this case, if F6TCNNQ molecules diffuse beneath the DN4T layer, “direct” charge transfer from HOPG to F6TCNNQ would occur, thereby raising the surface electrostatic potential. This newly defined surface electrostatic potential would act as the effective work function of the sample, also shifting the valence levels of any adsorbate above by an amount approximated by the work function change, as observed in earlier studies.^39,49^ This morphology is favored due to our observation that the spectral features at rather high F6TCNNQ nominal coverage still look like those at low coverage, just shifted in energy. If an appreciable amount of F6TCNNQ were adsorbed on top of DN4T and the CTCs to provide a homogeneous coverage (as implied by the large work function change), their spectral features should be significantly suppressed, because of the high surface sensitivity of UPS. Also, the interaction between HOPG and F6TCNNQ (integer electron transfer) is most likely stronger than between DN4T and F6TCNNQ (CTC formation only) probably due to the higher EA of FTCNNQ relative to the work function of HOPG (Figure 4c), This provides a driving force for the diffusion of excess F6TCNNQ directly toward the substrate. However, while EAs represent one important driving factor, another lies in the total electron densities of the molecules and the specific intermolecular interactions between them, which cannot be fully captured by the frontier orbital energies alone.

Consistent with this interpretation, essentially the same trend is observed when F6TCNNQ is deposited on a DN4T multilayer film of 25 Å nominal thickness (Figure S2, SI). The spectra in Figure S2 indicate that F6TCNNQ also interdiffuses beneath the DN4T multilayers, while only a smaller fraction forms CTCs at the topmost layer. Although the final spectrum in this case is somewhat broadened due to increased structural and electrostatic disorder in the thicker film, it closely resembles that of the F6TCNNQ/DN4T/HOPG interface. This suggests that CTCs are not forming only at the HOPG surface but also within thicker DN4T films, in agreement with our UV–vis absorbance spectra obtained from bulk F6TCNNQ:DN4T films. In addition, the fact that the S 2p level peak shifts less than the DN4T valence levels is likely because the S 2p core level has two components when F6TCNNQ is added: one from neutral DN4T molecules and one from those participating in the CTC. These two components cannot be resolved from our spectra, and thus the comparison of the S 2p level shift with DN4T before F6TCNNQ addition is probably simplified.

### 3.4 Estimation of the CTC Bandgap and Spectral Deconvolution

The deposition of F6TCNNQ onto DN4T/HOPG causes first the formation of CTCs followed by the above-described morphology and associated electrostatic potential landscape change at higher F6TCNNQ coverages, caused by the charge redistribution in the three-component interface. The formation of a DN4T:F6TCNNQ complex should be also facilitated by the molecular orientation of DN4T on HOPG. Scanning tunneling microscopy measurements reported in our previous work revealed that DN4T forms a well-ordered layer of flat-lying molecules on the HOPG surface.^30^ In such an adsorption geometry, the orientation of the exposed π electron density of the DN4T HOMO favors its wave function overlap with the F6TCNNQ LUMO. Interestingly, angle-dependent UPS measurements on the F6TCNNQ/DN4T/HOPG system (Figure S3, SI) show the photoelectron intensity of the valence states to peak at an off-normal emission angle,^30^ suggesting that molecules probably maintain their flat orientation post-F6TCNNQ deposition. Therefore, in the case of hybridization, the bandgap of the DN4T:F6TCNNQ complexes can be estimated using Hückel’s formula

1 $$IE_{CTC}\left(EA_{CTC}\right)=\frac{IE_{DN4T}+EA_{F6}}{2}\pm \frac{1}{2}E_{gap}^{CTC}$$

where IE_CTC_ (EA_CTC_) and *E*_gap_^CTC^ are the IE (EA) and the bandgap of the of the charge-transfer complex. Using the experimentally determined energy difference between H_DN4T_ (DN4T’s HOMO) and the H_C_ of the complex of 0.38 eV (Δ*E*_DN4T–CTC_), along DN4T’s IE (IE_DN4T_ = 6.2 eV) and F6TCNNQ EA (EA_F6_ = 5.6 eV), the bandgap can be calculated as

2 $$\Delta E_{DN4T-CTC}=IE_{CTC}-IE_{DN4T}=\frac{IE_{DN4T}+EA_{F6}}{2}-IE_{DN4T}+\frac{1}{2}E_{gap}^{CTC}$$

The resulting bandgap *E*_gap_^CTC^ of the complex is approximately 1.4 eV. This value correlates fairly well with the optical gap of 0.7 eV observed in the absorbance measurements (Figure 3a), considering an exciton binding energy of 0.7 eV, typical of Frenkel and charge-transfer excitons in organic systems, where electron–hole pairs are confined to small intermolecular distances.^50–52^ Given this bandgap and the position of the H_C_ peak in the UPS spectrum at about 1.3 eV, it is reasonable to assume that the LUMO of the complex is then pinned at the Fermi level. This is further supported by the slightly larger SECO shift of 1.02 eV observed for the F6TCNNQ/DN4T/HOPG interface (Figure 4a), compared to the smaller shift of 0.92 eV for F6TCNNQ directly on HOPG (Figure S1, SI), and to the case of thicker DN4T films covered with F6TCNNQ (Figure S2, SI). In both cases, the electronic levels shift by ∼0.95 eV, but at the F6TCNNQ/DN4T/HOPG interface an additional ∼0.1 eV SECO shift is detected. This extra shift may be attributed to pinning of the CTC LUMO, associated with interfacial charge rearrangements and formation of interfacial dipoles, thereby further contributing to the overall SECO shift in the same direction.

Under the above hypothesis, the final photoelectron spectrum of F6TCNNQ/DN4T/HOPG (14 Å F6TCNNQ) system labeled (1) in Figure 7 can be deconvoluted into three distinct components: (i) The neutral DN4T component, labeled as (2) and electrostatically shifted by −0.95 eV toward the Fermi level; (ii) The F6TCNNQ anions component, marked as (3), (iii) the spectral component associated with the electronic density of states of the DN4T:F6TCNNQ complexes indicated as (4) and obtained by subtracting the components (2) and (3) from the total spectrum (1). It was not necessary to subtract an additional contribution from the neutral F6TCNNQ signal obtained at much higher F6TCNNQ coverages (Figure S1, SI). This can be attributed to the pronounced island growth and pillar-like aggregate formation that have been previously reported.^32,44,53^ Since these aggregates grow predominantly in the vertical direction, they cover only a small fraction of the surface area and therefore are expected to contribute less to the overall photoelectron signal.

**Figure 7:**
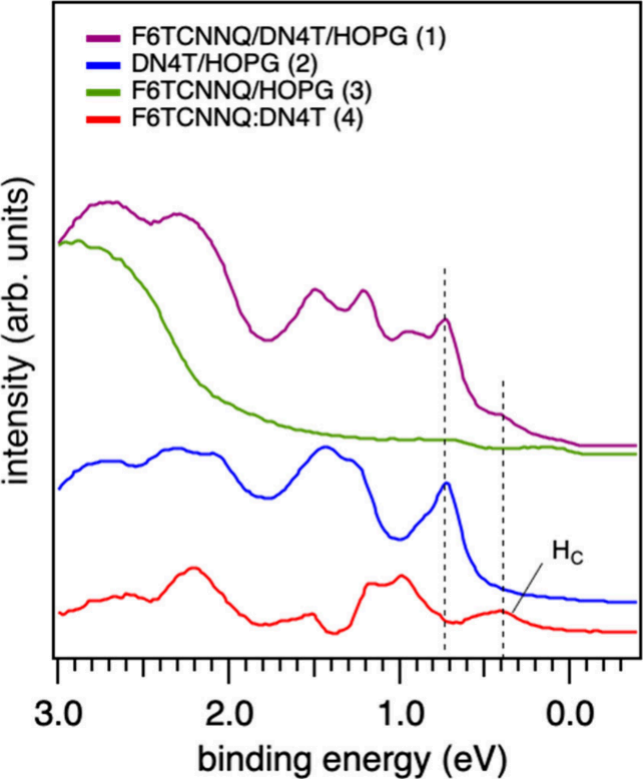
Subtraction analysis of UPS valence spectra. The purple curve (1) corresponds to the valence spectrum of the F6TCNNQ/DN4T/HOPG system (14 Å F6TCNNQ), and the blue curve (2) is the valence spectrum of the neutral DN4T on HOPG. The green spectrum (3) is the valence spectrum of the F6TCNNQ deposited on HOPG, corresponding to the F6TCNNQ anions. The red curve (4) is obtained after subtracting (2) and (3) from (1) and should correspond to the valence spectrum of the F6TCNNQ:DN4T complex. The dashed lines indicate the position of the HOMO of the neutral DN4T and the position of the hybrid HOMO of the complex, H_C_.

A similar analysis for the F6TCNNQ/isoDN4T/HOPG system was not practicable. As shown in Figure S4 (SI) the spectra of isoDN4T after F6TCNNQ deposition change dramatically with very little spectral detail. Immediately upon F6TCNNQ deposition the spectra seemingly shift and become significantly broader, rendering the features much less distinguishable and making it difficult to quantify valence shifts or resolve individual components. This observation aligns with the absorbance measurements in Figure 3b, where the stronger interaction between isoDN4T and F6TCNNQ suggests a higher concentration of F6TCNNQ:isoDN4T complexes.

As with DN4T, electron transfer from isoDN4T to F6TCNNQ is also energetically unfavorable due to the much higher IE of isoDN4T (6.2 eV) compared to the EA of F6TCNNQ. However, electron transfer from HOPG to F6TCNNQ is still expected. This is inferred from the SECO shift by 1.1 eV, essentially reflecting the shifts observed in both F6TCNNQ/HOPG and F6TCNNQ/DN4T/HOPG and also from the presence of spectral features at the Fermi level, again likely associated with F6TCNNQ anions.

Taken together, these results suggest that while CTC formation with DN4T does not significantly alter the surface electrostatic potential and thereby the work function at low F6TCNNQ coverages, the stronger interaction of isoDN4T with F6TCNNQ drives abrupt structural rearrangements and induces enhanced electrostatic disorder across the film. This homogeneous disorder can account for the immediate work function shift observed in the isoDN4T system, even without substantial dopant interdiffusion. As a result, the pronounced electrostatic disorder and the associated broadening of the photoelectron spectra make it difficult to resolve the HOMO contributions and the respective shifts, preventing also a reliable quantitative decomposition of the valence region as performed for the DN4T case.

We finally note that previous theoretical studies have explored the potential for integer charge transfer in host-dopant systems, driven by favorable intermolecular polarization effects that could sufficiently reduce the host-dopant energy barrier for intermolecular charge transfer. For instance, Privitera *et al*. demonstrated that such effects can arise from the interaction of quadrupole moments between host and dopant molecules.^18^ If this were the case in the systems presently investigated, the formation of positively charged DN4T molecules would result in almost pristine DN4T spectral features with a much higher IE due to the stronger Coulombic attraction of the electrons in the ionized final state. However, our observations contrast with this scenario as the H_C_ feature emerges at lower binding energies giving a smaller IE and the H_DN4T_ feature shifts with the vacuum level, yielding a constant IE.^48,54^ All these results indicate that DN4T is not involved in electron transfer processes and no evidence of positive DN4T polarons is observed in our UPS, XPS, and optical absorbance measurements.

## 4 Conclusions

In this study, we investigated the intermolecular interactions and electronic properties of F6TCNNQ/DN4T surface layers deposited on HOPG and compared them with the structurally similar F6TCNNQ/isoDN4T/HOPG system, using *in-situ* photoelectron spectroscopy. Complemented by optical absorbance measurements our findings provide clear evidence of strong host-dopant interaction between (iso)­DN4T and F6TCNNQ, involving CTC formation in both bulk and interfacial mixtures. Our results further reveal a complex adsorption process. Once CTC formation saturates, additional F6TCNNQ molecules diffuse to the HOPG surface, resulting in direct electron transfer from HOPG to F6TCNNQ which raises the sample work function and shifts the electronic states of both DN4T and the F6TCNNQ:DN4T CTCs by the same amount. This charge redistribution could occur via two configurations. In one scenario, F6TCNNQ adsorbs on top of the DN4T and CTC layers, resulting in charge transfer between HOPG and the F6TCNNQ layer—a situation analogous to a dielectric (DN4T and CTCs) placed within a parallel-plate capacitor formed by HOPG (positive plate) and F6TCNNQ anions (negative plate). Alternatively, F6TCNNQ might interdiffuse beneath the DN4T layer, where direct charge transfer between HOPG and F6TCNNQ would take place. Moreover, our evidence indicates that F6TCNNQ interacts more strongly with isoDN4T than with DN4T. This stronger interaction in the isoDN4T system leads to significant changes in the DOS observed by UPS, so much so that the original isoDN4T DOS is no longer recognizable, in contrast to DN4T. These findings underscore how subtle structural variations such as those in isomeric molecules can dramatically influence host–dopant interactions. To fully rationalize why F6TCNNQ binds and perturbs isoDN4T more strongly than DN4T, complementary atomic-scale microscopy, advanced photoelectron spectroscopies, and first-principles calculations will be necessary to pinpoint preferential locations of F6TCNNQ on DN4T and isoDN4T, adsorption geometries, film morphology, and the influence of frontier-orbital distributions, efforts that lie beyond the scope of this study. Future studies that explore different substrates, organic semiconductors, and molecular dopants, as well as the careful design of multicomponent systems, may reveal new strategies for surface modification and pathways to optimize charge injection and transport in organic electronic devices.

## Supplementary Material



## Notes

Thickness-dependent UPS spectra of pristine F6TCNNQ; angle-dependent UPS spectra of the F6TCNNQ/DN4T/HOPG system; thickness-dependent UPS spectra of the F6TCNNQ/isoDN4T/HOPG system; UPS spectra of F6TCNNQ deposited on DN4T multilayer films (25 Å), showing interdiffusion and CTC formation beyond the monolayer regime (PDF)
