# Supplementary material for: Molecular Doping Induced Charge Transfer Complex Formation and Interfacial Dopant Interdiffusion on Graphite
Source: J Phys Chem C Nanomater Interfaces. 2025 Dec 4;129(50):22120–9. doi: 10.1021/acs.jpcc.5c05680 (PMC12720229; doi:10.1021/acs.jpcc.5c05680)
Supplement: Supplementary file 1 [file jp5c05680_si_001.pdf]

Supporting information

for

Publication

Molecular Doping Induced Charge Transfer Complex Formation and Interfacial Dopant Inter-diffusion on Graphite

Christos Gatsios[1], Andreas Opitz[1], Patrick Amsalem[1], Thorsten Schultz[1,2], Remy Jouclas[3], Yves Geerts[2,4] and Norbert Koch[1,2,*]

[1]Institut für Physik & Center for the Science of Materials Berlin (CSMB), Humboldt-Universität zu Berlin, 12489 Berlin, Germany

[2]Helmholtz-Zentrum Berlin für Materialien und Energie GmbH, 12489 Berlin, Germany

[3]Laboratoire de Chimie des Polymères, Faculté des Sciences, Université Libre de Bruxelles (ULB), Boulevard du Triomphe, CP 206/01, Bruxelles 1050, Belgium

[4]International Solvay Institutes for Physics and Chemistry, Université Libre de Bruxelles (ULB), Boulevard du Triomphe, CP 231, Bruxelles 1050, Belgium

*E-Mail: norbert.koch@physik.hu-berlin.de

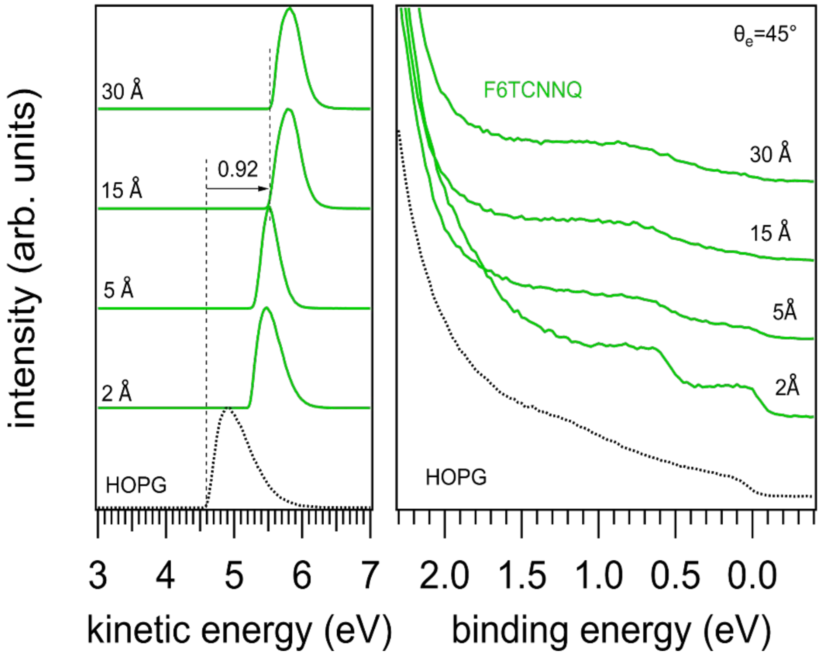

Supplementary Figure S1 Thickness dependent UPS measurements of F6TCNNQ on HOPG obtained at a 45° off-normal emission angle. The left graph shows the SECO region whereas the right graph shows the valence region. After deposition F6TCNNQ the SECO abruptly shifts, showing a total shift of 0.92 eV towards higher kinetic energy due to electron transfer at the interface. At the same time, two new features appear at the valence spectrum. The lower binding energy peak corresponds to the relaxed LUMO level while the higher binding energy is the relaxed HOMO of the negatively charged F6TCNNQ.

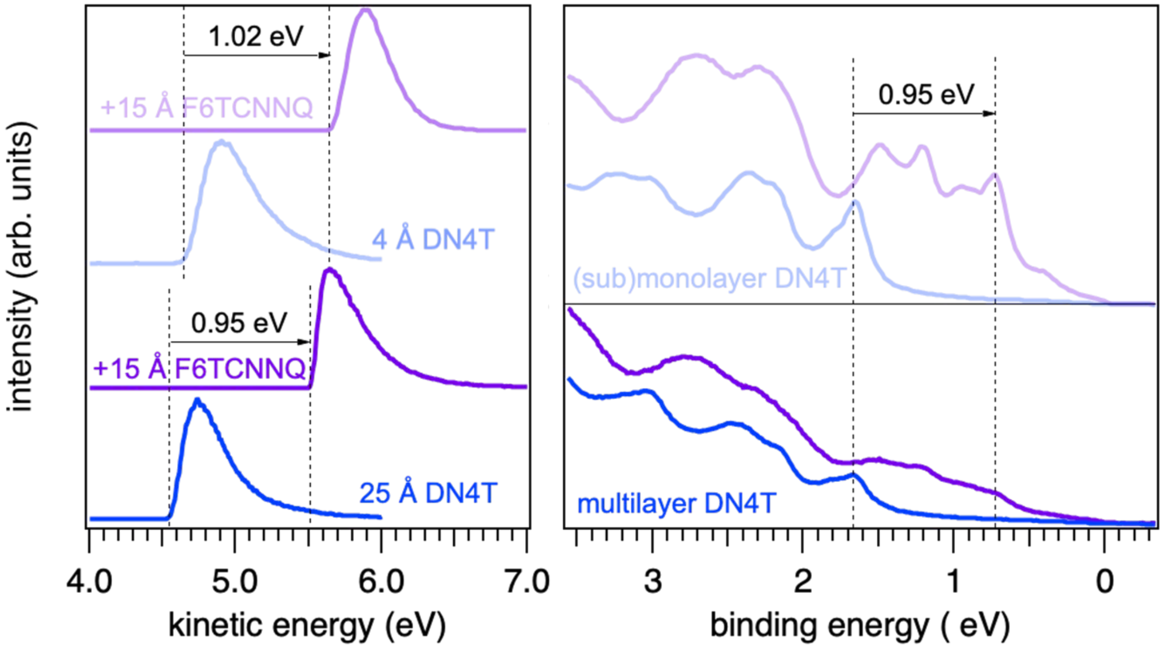

Supplementary Figure S2 Secondary electron cutoff (SECO, left) and valence spectra (right) for F6TCNNQ deposited on DN4T films of different thicknesses. The lower solid curves in each panel correspond to a DN4T multilayer of 25 Å nominal thickness covered with 15 Å of F6TCNNQ, while the paler solid curves above correspond to a sub-monolayer DN4T film of 4 Å nominal thickness on HOPG covered with 15 Å of F6TCNNQ. In the interface regime (sub-monolayer DN4T), the SECO shifts by 1.02 eV, whereas in the thicker DN4T multilayer regime the SECO shift is 0.95 eV. In both cases, the electronic levels shift by 0.95 eV. The additional ~0.07 eV shift of the SECO at the interface compared to the multilayer regime may reflect LUMO level pinning, which is expected to be more pronounced at the interface due to the formation of interfacial dipoles associated with charge rearrangements. The black dividing line in the right panel separates the spectra of multilayer DN4T (bottom) from those of (sub)monolayer DN4T on HOPG (top).

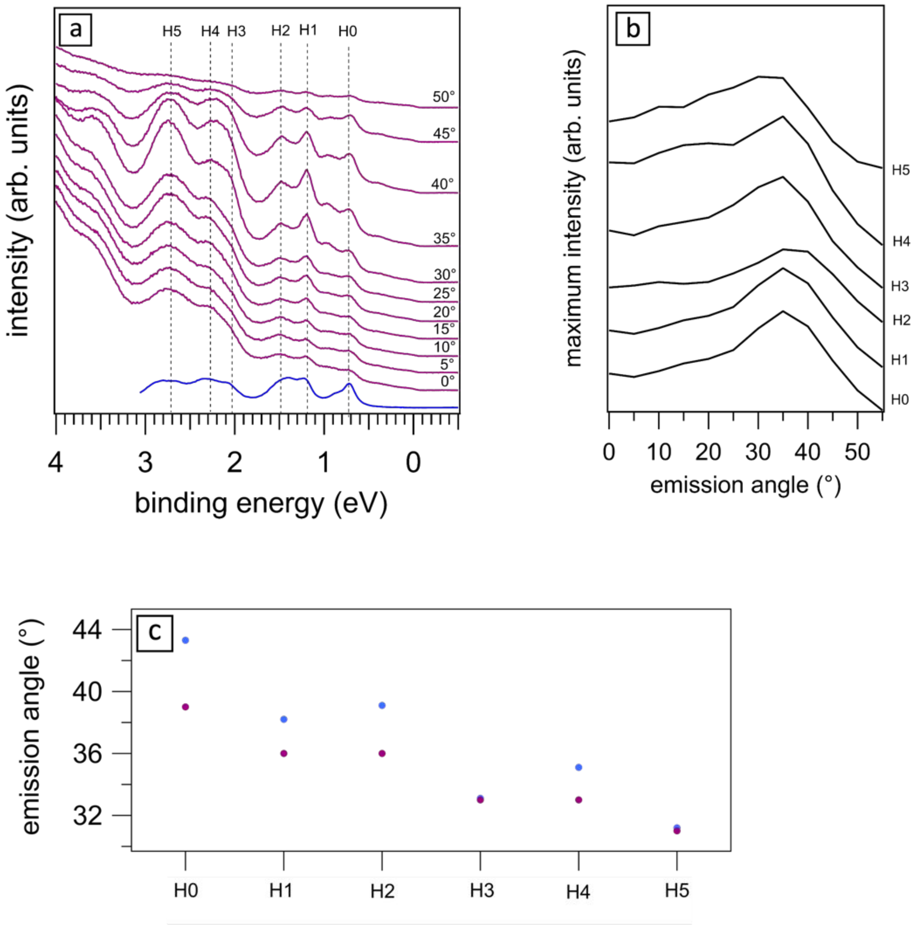

Supplementary Figure S3 (a) Angle-dependent UPS measurements of F6TCNNQ/DN4T/HOPG system. The purple curves indicate spectral evolution of the HOPG/DN4T/F6TCNNQ varying the emission angle, while the blue curve allows comparison with the DN4T spectrum at 45°. The dashed lines show the positions of peaks that match with the respective peaks of DN4T. (b) The maximum intensity of each peak is plotted as a function of the emission angle. The intensity of all peaks maximizes at an off-normal emission angles 35-40°. (c) Comparison of the angle at maximum intensity in the two systems, namely DN4T/HOPG and F6TCNNQ/DN4T/HOPG. The blue dots correspond to the DN4T/HOPG and the purple dots correspond to the F6TCNNQ/DN4T/HOPG. In both cases the angle at the maximum intensity of each peak follows the same trend, suggesting that DN4T molecules maintain the predominantly flat orientation after evaporation of F6TCNNQ.

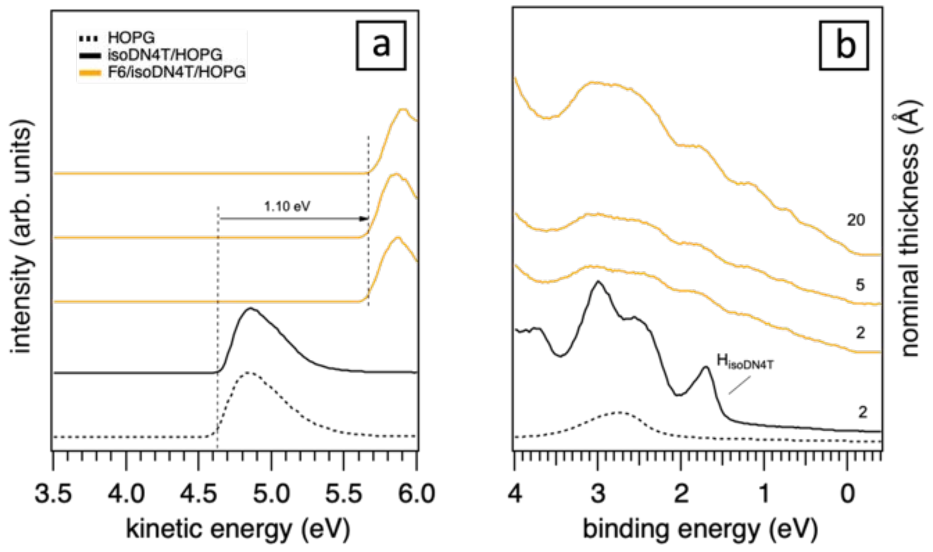

Supplementary Figure S4 Thickness dependent UPS measurements. (a) SECO and (b) valence region spectra, recorded at an off-normal emission angle of 45°. The dashed black curve corresponds to the HOPG substrate, while the solid black curve represents 2 Å of isoDN4T deposited on HOPG corresponding to (sub)monolayers of isoDN4T. The yellow curves show the evolution of the SECO and valence region after successive deposition of F6TCNNQ. Following the deposition of F6TCNNQ, we observe a shift of the SECO by 1.1 eV, which can be attributed to a change of the surface electrostatic potential due to electron transfer from HOPG to F6TCNNQ. Additionally, the valence spectrum of isoDN4T seemingly shifts simultaneously but becomes essentially featureless, in stark contrast to DN4T. This is likely due to stronger electronic interaction and enhanced wavefunction overlap between F6TCNNQ and isoDN4T. The significant broadening may also indicate an increased structural disorder on the surface compared to DN4T. Indeed, a larger concentration of isoDN4T:F6TCNNQ complexes may disrupt the ordered arrangement of isoDN4T molecules on the surface of HOPG, thereby increasing electrostatic disorder in the electronic system.
